# miR-500 promotes cell proliferation by directly targetting LRP1B in prostate cancer

**DOI:** 10.1042/BSR20181854

**Published:** 2019-04-05

**Authors:** Zhaoli Zhang, Ran Cui, Hui Li, Jinlong Li

**Affiliations:** 1 Department of Pharmacy, Nanjing Second Hospital, Nanjing University of Chinese Medicine, Nanjing 210003, China; 2 Department of Pediatrics, The Taizhou People’s Hospital, Taizhou 225300, China; 3 Department of Laboratory Medicine, Nanjing Second Hospital, Nanjing University of Chinese Medicine, Nanjing 210003, China

**Keywords:** LRP1B, miR-500, prostate cancer, proliferation

## Abstract

Accumulating evidence suggests that miRNAs play a crucial role in the development of prostate cancer (PC); however, the role of miR-500 in PC remains poorly understood. The data presented here reveal abnormal increases in miR-500 expression in PC tissues and cell lines. Suppression of miR-500 expression significantly inhibited the proliferation of PC-3 and LnCap cells and was negatively regulative with low-density lipoprotein receptor-related protein 1B (LRP1B). Increased cell cycle arrest at the G1 stage and decreased protein expression of cyclinD1 and CDK2 was observed in response to miR-500 knockdown in PC-3 and LnCap cells, in combination with LRP1B overexpression. LRP1B was identified as a target of miR-500 and was significantly decreased in PC tissues. Taken together, these findings demonstrate that miR-500 plays an important role in the proliferation of PC cells via the inhibition of LRP1B expression.

## Introduction

Prostate cancer (PC) is one of the most common and aggressive human malignancies, and remains a significant cause of mortality worldwide [1]. Recent advances in adjuvant chemotherapy, radiotherapy, and tumor resection have provided significant improvements in patient outcomes [2], with new strategies, such as gene-targetted therapies, receiving considerable attention in recent years [3]. However, the prognosis and 5-year survival rates of PC patients remain poor, highlighting the need for a better understanding of the molecular mechanisms underlying tumorigenesis in PC.

miRNAs are small, non-coding RNAs, typically 20–25 nts in length, which inhibit post-transcriptional gene expression by sequence-specific interaction with the 3′ non-coding region of homologous mRNA. [4]. Their role in tumorigenesis is well documented, beginning with the first report by Calin et al. in 2002 [5]. Since then, it has been found that miRNA is specifically expressed in a variety of tumors and plays an important role in tumor proliferation [6], differentiation, invasion and metastasis [7], and treatment [8]. Recent findings have shown that miRNAs play a role involved in virtually all biological processes, including the occurrence, diagnosis, treatment, and prognosis of cancer [9]. Amongst the various miRNA molecules described to date, miR-500 has been reported to be significantly up-regulated and acts a role as a promoter in several cancer types, including hepatocellular carcinoma [10], ovarian cancer [11], and non-small cell lung cancer [12]. Increasing evidence also suggests that miR-500 plays a key role in cancer development [13]; however, the mechanisms by which miR-500 confers these effects in PC is not well understood.

Here, we provide the first description of miR-500 expression in PC normal tissues. Functional characterizations of miR-500 in the PC-3 and LnCap cell lines were also explored to understand its role in PC more fully.

## Material and methods

### Patient tissue specimens

We get the prior approval as well as the written informed consents from 35 patients and their PC specimens and adjacent non-tumor tissues were collected from the same patient. All clinical samples were histologically examined by pathologists from June 2015 to April 2017 in The Second Hospital of Nanjing, Nanjing University of Chinese Medicine. The research has been carried out in accordance with the World Medical Association Declaration of Helsinki. The present study was approved by the Institutional Research Ethical Committee of The Second Hospital of Nanjing.

### Cell line culture and transfection

Human PC cell lines (PC-3, Du145, and LnCap), the human prostate epithelial cell line RWPE-1 was purchased from Shanghai Institute of Cell Bank (Shanghai, China) and cultured in DMEM/F12 medium containing with 10% fetal bovine serum (FBS), 100 IU/ml penicillin, and 100 μg/ml streptomycin at 37°C under 5% CO_2_. miR-500 inhibitor, miR-500 inhibitor negative control, pcDNA3.1 containing sequence of low-density lipoprotein receptor-related protein 1B (LRP1B) or pcDNA3.1 negative control were transfected using Lipofectamine 2000 following the manufactures’ instructions.

### Quantitative real-time PCR

Total tissue and cell RNA was extracted using TRIzol reagent (Invitrogen) according to the manufacturer’s instructions. Six microliter extracted RNA were reverse transcribed using the PrimeScript™ RT reagent Kit with gDNA Eraser (TAKARA) according to the provider’s protocol. Quantitative PCR was performed using SYBR^®^ Green Real time PCR Master Mix (TAKARA) in the StepOnePlus Real-Time PCR System (Applied Biosystems). The expression of mRNA or miRNA was normalized using GAPDH and U6 as endogenous control, respectively. The specific primers were as follows: LRP1B forward, 5′- TTT CTC CTC GCC TTA CTC ACT -3′ and reverse, 5′- CAC ACA ACT GCT GAT CTC GGT -3′; GAPDH forward, 5′- AGT GCC AGC CTC GTC TCA TA -3′ and reverse, 5′- GGT AAC CAG GCG TCC GAT AC -3′. miR-500 forward, 5′-ATC CTT GCT ACC TGG GTG AGA-3′, reverse, 5′-GCT CTC GCT CTC AGA ATC CTT-3′, U6 forward, 5′- CTC GCT TCG GCA GCA CA -3′ and reverse, 5′- AAC GCT TCA CGA ATT TGC GT -3′. All the samples were amplified in triplicate and each experiment was repeated three times.

### Western blot

After rinsing with PBS, the PC cells were added ice-cold RIPA buffer containing protease inhibitor cocktail (1:100, Sigma). After determination of protein concentration, primary antibodies for western blot were anticyclinD1 (dilution, 1:1000; #2922; Cell Signaling, CA, U.S.A.), anti-CDK2 (dilution, 1:1000; #2546; Cell Signaling, CA, U.S.A.), anti-LRP1B (dilution, 1:1000; H00053353-A01; Abnova), and anti-GAPDH (dilution, 1:3000; #2118; Cell Signaling, CA, U.S.A.). HRP-conjugated anti-rabbit IgG (1:5000) antibody was used as the secondary antibody. The specific bands were visualized with ECL reagent and captured by G: BOX Chemi XT4 (Syngene, U.S.A.) and then visualized with Quantity One software 4.6.2.

### Luciferase assays

After cultured in 24-well plates, the PC cells (PC-3 and LnCap) were cotransfected with 10 ng of pRL-TK wild-type (wt) or mutant (mut) LRP1B reporter plasmid, 100 ng firefly luciferase reporter plasmid, and 10 pmol miR-500 mimic or NC following the manufacturer’s protocol. The firefly luciferase activity was measured and normalized to Renilla signals at 48 h post-transfection.

### Cell proliferation assay

Cells (10^4^/96-well plate) were plated and cultured. Then the cell proliferation rate was detected at 24, 48, 72, and 96 h. Twenty microliter of MTT was added following a 4 h incubation at 37˚C. Subsequently, 150 µl DMSO was then added and incubated for 10 min at room temperature. The absorbance value was determined by using the XT-96DJ ELISA analyzer at 570 nm.

### Colony formation assay

The colony formation assay was conducted as previously described [14]. Briefly, 48 h after cell transfection, 100 μl cells (5 × 10^4^ cells/ml) was added into the six-well plates. Total 12 days later, colonies were stained with 0.1% crystal violet (Sigma-Aldrich Co.) in 20% methanol for 15 min. Colony number of visible colonies was counted. Each experiment was repeated in triplicate.

### Cell cycle analysis

After washed twice with PBS, PC-3, and LnCap cells were resuspendedand fixed in 70% ethanol overnight at 4˚C. Subsequently, PC-3 and LnCap cells added the PI for incubation 30 min. Cell cycle was then analyzed using BD™ LSRII flow cytometry system with CellQuest Pro software version 5.1 (BD Biosciences, Franklin Lakes, NJ, U.S.A.).

### Statistical analysis

All values were expressed as mean ± S.E.M. and analyzed by oneway ANOVA followed by Tukey’s *post hoc* test amongst groups using Statistical Product and Service Solutions (SPSS) (Version 17.0). The *P*-values less than 0.05 were considered as statistically significant difference between groups.

## Results

### miR-500 is up-regulated and LRP1B is down-regulated in PC tissues and cells

**Figure 1:**
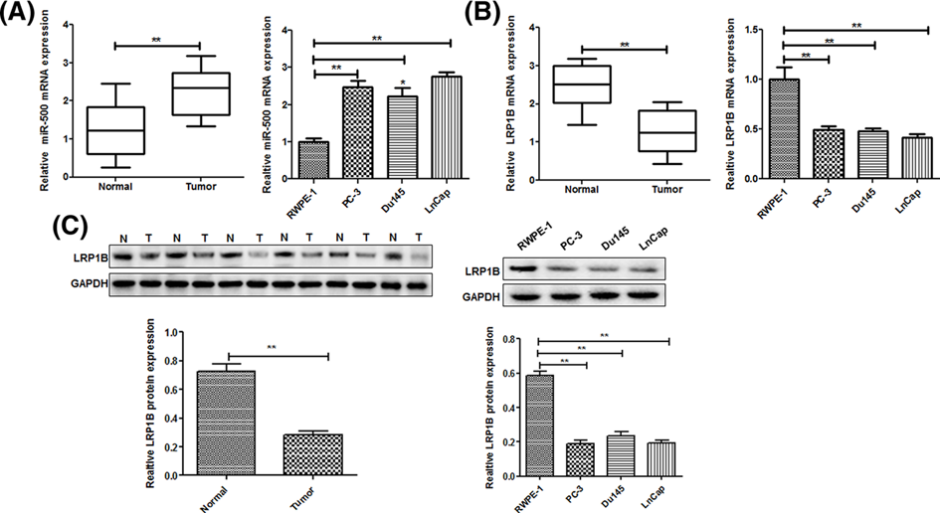
miR-500 is up-regulated and LRP1B is down-regulated in prostate cancer tissues and cells Expressions of miR-500 (**A**) and LRP1B (**B**) in human prostate cancer tissues, adjacent non-tumor tissues, and human prostate cancer cell lines PC-3, Du145, and LnCap cells, and a non-cancer cell line RWPE-1 were detected by quantitative real-time PCR. (**C**) Protein expression of LRP1B in human prostate cancer tissues, adjacent non-tumor tissues, and human prostate cancer cell lines PC-3, Du145, and LnCap cells, and a non-cancer cell line RWPE-1 were detected by western blot.*P<0.05, **P<0.01.

In order to confirm whether miR-500 was involved in the regulation of PC tumorigenesis, we detected the miR-500 expression in 35 paired of PC tissues and adjacent non-tumor tissue by using qRT-PCR analysis. Interestingly, PC tissues showed a frequently increased expression of miR-500 compared with the matched non-tumor tissues. Consistent with those results the miR-500 levels in PC cell lines, namely PC-3, Du145 and LnCap, were determined to be up-regulated in PC cell lines compared with that of human prostate epithelial cell line RWPE-1 (Figure 1A). Meanwhile, the mRNA (Figure 1B) levels and protein expression (Figure 1C) of LRP1B were down-regulated in the PC tissues and PC cell lines. Consequently, miR-500 was significantly up-regulated and LRP1B was down-regulated in PC.

### Knockdown of miR-500 inhibits the proliferation of PC cells

**Figure 2:**
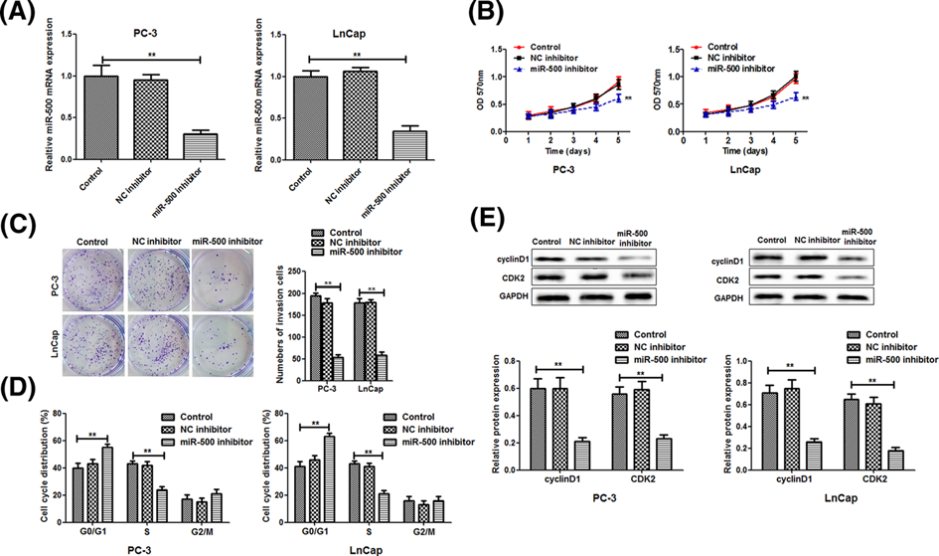
miR-500 inhibitor suppresses celll proliferation by regulating the cell cycle miR-500 levels in PC-3 and LnCap cells transfected with NC inhibitor or miR-500 inhibitor were determined by reverse-transcription quantitative PCR analysis (**A**). The MTT assay (**B**) and colony assay (**C**) were performed to examine the cell proliferation. Flow cytometry was used to examine the cell cycle distribution (**D**). The protein expression of cyclinD1 and CDK2 was detected using the western blot (**E**). **P<0.01.

Next, PC-3 and LnCap cells were cotransfected with miR-500 inhibitor. First, we determined the transfection efficiency of miR-500 inhibitor by qRT-PCR analysis, showing a significant reduce of miR-500 levels in PC-3 and LnCap cells compared with the negative control group (Figure 2A). Moreover, we found knockdown of miR-500 could significantly inhibit the proliferation of PC-3 and LnCap cells compared with that of control group according the MTT results (Figure 2B) and colony formation assay (Figure 2C), suggesting that knockdown of miR-500 inhibited the proliferation of PC cells.

Moreover, we detected the cycle distribution by using flow cytometry in PC-3 and LnCap cells. It was determined that miR-500 knockdown resulted in a marked cell cycle arrest in G1 phase of PC-3 and LnCap cells (Figure 2D). In addition, the expression of cyclinD1 and CDK2, which are the two main regulators in cell cycle, were significantly reduced in the miR-500 inhibitor group (Figure 2E), which may contribute to strengthen the inhibition of PC cell proliferation by miR-500 knockdown.

### miR-500 directly targets LRP1B in PC cells

**Figure 3:**
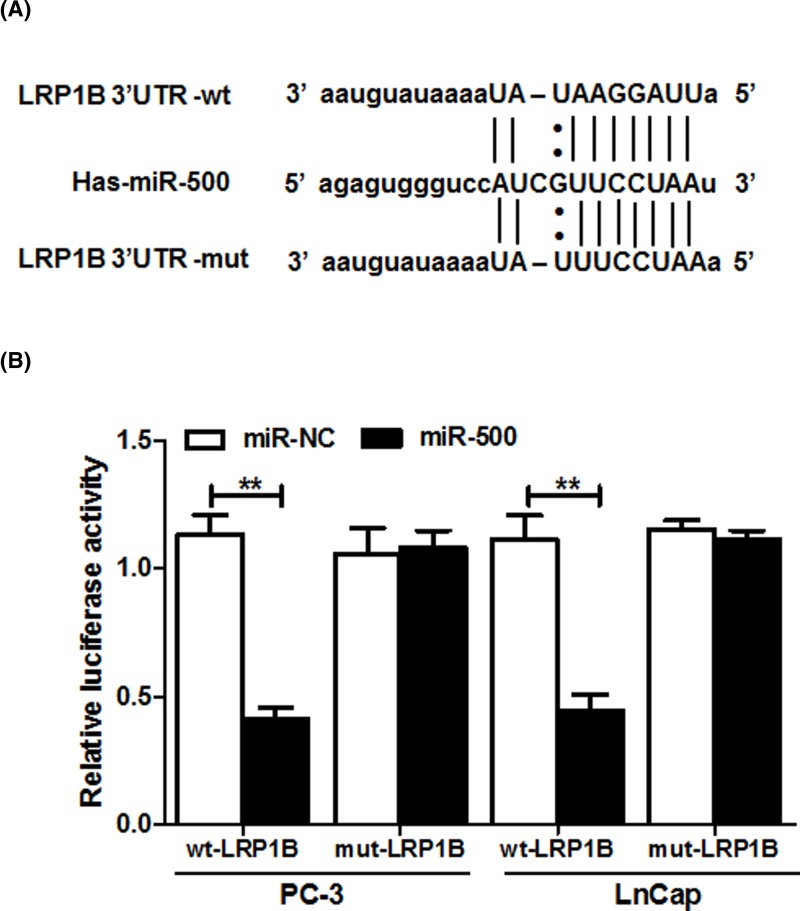
LRP1B is a direct target of miR-500 in prostate cancer cells (**A**) Putative wild-type or mutant miR-500-binding sites in the LRP1B mRNA 3′ untranslated region. (**B**) Relative luciferase activity of PC-3 and LnCap cells cotransfected with the constructed luciferase reporters (pGL3-LRP1B-wild type and pGL3- LRP1B-mutated), pRL-TK vectors, and miR-500 or the miR-NC. **P<0.01.

**Figure 4:**
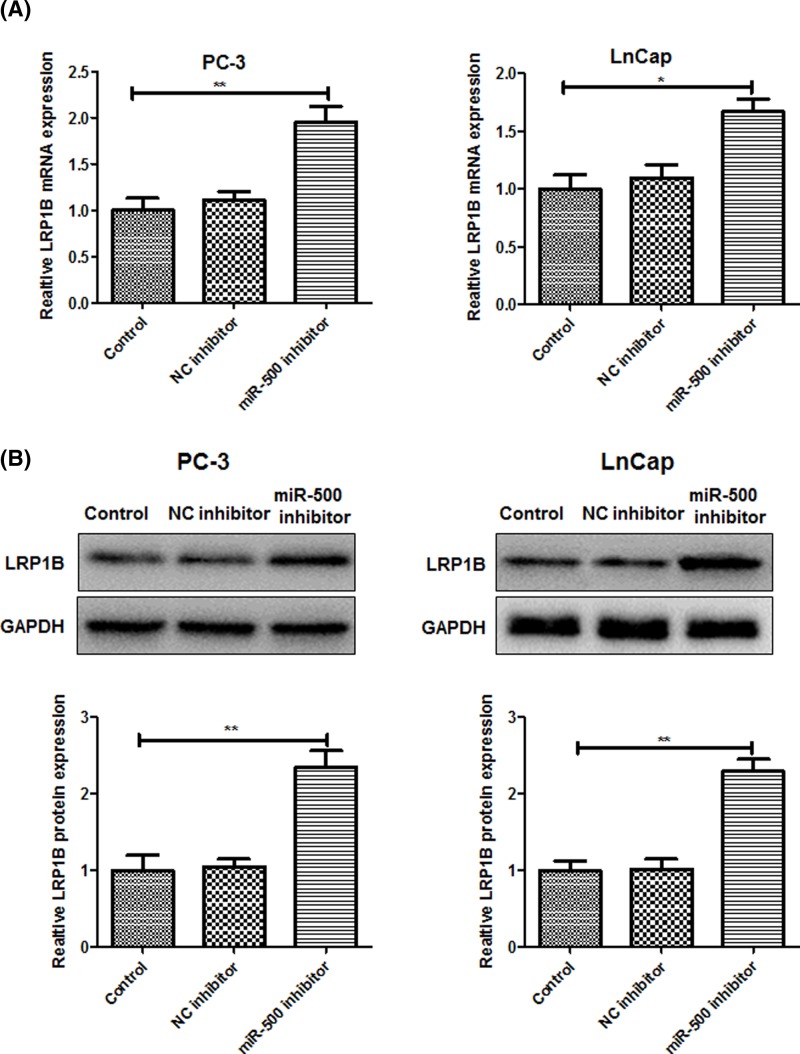
miR-500 negatively regulates LRP1B (**A**) The mRNA levels of LRP1B in PC-3 and LnCap cells transfected with NC inhibitor or miR-500 inhibitor, respectively, were determined by reverse-transcription quantitative PCR analysis. (**B**) Western blot analysis was used to examine the protein levels of LRP1B. *P<0.05, **P<0.01. miR-500 negatively regulates LRP1B

Using the Targetscan software, it is indicated that LRP1B is a putative target gene of miR-500 (Figure 3A). Meanwhile, luciferase assay indicated that miR-500 overexpression significantly suppressed the luciferase activities of reporter, but silence of miR-500 markedly increased the luciferase activities. However, but the luciferase activity of the mutant reporter was unaffected (Figure 3B). Importantly, in order to further to determine the target of miR-500 with LRP1B, the mRNA and protein expression of LRP1B were detected after miR-500 inhibitor transfection. According to the results of RT-qPCR (Figure 4A) and western blot (Figure 4B), those results confirmed that the LRP1B expression was significantly increased in PC-3 and LnCap cells transfected with miR-500 inhibitor, proving that LRP1B was identified as a target gene of miR-500 in PC cells.

### LRP1B is involved in the miR-500-mediated proliferation of PC cells

**Figure 5:**
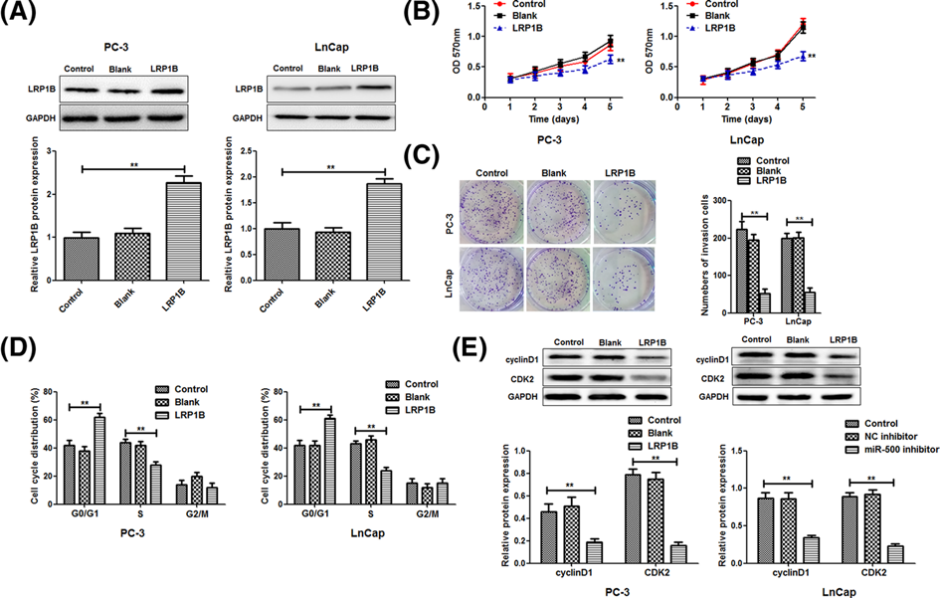
LRP1B inhibits the cell proliferation by regulating the cell cycle (**A**) Western blot analysis was used to examine the protein levels of LRP1B in PC-3 and LnCap cells transfected with blank pcDNA3.1 vector or pcDNA3.1-LRP1B open reading frame plasmid, respectively. The MTT assay (**B**) and colony assay (**C**) were used to examine the cell proliferation. (**D**) Flow cytometry was used to examine the cell cycle distribution. (**E**) The protein expression of cyclinD1 and CDK2 was detected using the western blot. **P<0.01.LRP1B inhibits the cell proliferation by regulating the cell cycle

From the results above, we determined that knockdown of miR-500 could increase the LRP1B expression, accompany with a cell cycle arrest at G1 stage in PC cells, speculating that LRP1B may play a downstream effector role in miR-500-mediated PC cell proliferation. The protein expression of LRP1B in PC-3 and LnCap cells was markedly increased compared with the control group after pcDNA3.1-LRP1B ORF plasmid transfection in PC-3 and LnCap cells (Figure 5A).

In addition, MTT assay (Figure 5B) and colony formation assay (Figure 5C) date showed that overexpression of LRP1B also inhibited PC-3 and LnCap cells proliferation, identical with the effect of miR-500 knockdown. In addition, similar to the effect of miR-500 knockdown, flow cytometry analysis showed that up-regulated LRP1B expression contributed to the cell cycle arrest at the G1 stage in PC cells (Figure 5C). In addition, the expression of cyclinD1 and CDK2 were significantly reduced in the overexpression LRP1B group (Figure 5E). These results indicated that LRP1B was indeed participated in the miR-500-mediated PC cell proliferation.

## Discussion

A large number of studies have demonstrated a role for miRNAs in the development and proliferation of various tumors due to their regulation of tumor growth, apoptosis, differentiation, angiogenesis, invasion, and metastasis. miRNAs have been shown to act as both oncogenes or tumor suppressors, including several miRNAs regulating the various mechanisms underlying PC pathogenesis [15]. In the present study, the role and molecular mechanisms of miR-500 in the regulation of PC was explored. Our data revealed a significant decrease in miR-500 expression in PC tissues. In contrast, overexpression of miR-500 strongly attenuated cell proliferation in PC cells, showing that miR-500 may play an important role in regulating PC pathogenesis. Targetted knockdown of miR-500 suppressed cell proliferation in PC-3 and LnCap cells, consistent with *in vitro* experiments showing cell cycle arrest at the G1 stage. As the two main regulators in cell cycle, the protein expression of cyclinD1 and CDK2 were detected by western blot. The results showed that miR-500 inhibitor could significantly decrease the levels of cyclinD1 and CDK2, indicating that miR-500 plays an important role in the cell cycle redistribution in PC-3 and LnCap cells. Luciferase assays identified LRP1B as a direct target of miR-500, implicating miR-500 as an important mediator of cell proliferation in PC. Finally, we showed that LRP1B expression was significantly down-regulated in PC tissues relative to matched adjacent non-tumor tissues, showing that increased miR-500 expression may be the result of LRP1B inhibition in PC.

Emerging evidence suggests that miR-500 functions as either an oncogene or tumor suppressor depending on the type of cancer [16]. These effects are highly dependent on its various expression levels in certain tumors, along with the function of target genes, including those regulating proliferation, invasion, and cell migration [17]. For example, Yamamoto, et al. reported that miR-500 is abundantly expressed in the sera and tumor tissues of hepatocellular carcinoma (HC) patients, but could be restored to baseline levels following tumor resection [18]. Various studies have also suggested that increased miR-500 expression may be associated with poor clinical outcomes in gastric tumors. In addition, miR-500 sustains nuclear factor-kappaB (NF-κB) activation and induces gastric cancer cell proliferation and resistance to apoptosis [17]. Despite these observations, the exact role, whether pro- or antimetastasis, of miR-500 in PC remains poorly understood. Here, we observed significant increases in miRNA-500 expression in PC tissues and cell lines, which may contribute to malignancy in PC. To verify the hypothesis, PC-3 and LnCap cells were transfected with a miR-500 inhibitor, revealing potent suppression of proliferation in PC-3 and LnCap cells. Taken together, these observations suggest that miR-500 is an important mediator of oncogenesis in PC.

LRP1B, a member of the low density lipoprotein (LDL) receptor family, is identified as a new candidate tumor suppressor gene [19,20]. This gene plays multiple roles in normal cell function and development [21], probably mediated by its binding to extracellular ligands. This gene has been found to be inactivated in various malignancies, including urothelial cancer [22], esophageal carcinoma [23], ovarian cancer [24], glioblastoma [25], gastric cancer [26], thyroid cancer [27], and lung carcinoma [28]. Although LRP1B has been identified as a tumor suppressor in several cancer types, its expression pattern and biological function in PC remain poorly understood. Here, we explored the expression, functions, and mechanism of action of LRP1B in PC. Using a luciferase reporter assay, we showed that LRP1B was a direct target of miR-500 in PC. Moreover, miR-500 knockdown significantly enhanced LRP1B protein levels in PC-3 and LnCap cells, while LRP1B overexpression markedly inhibited the proliferation in PC-3 and LnCap cells, proving that LRP1B is an important downstream effector of miR-500 in PC proliferation. Interestingly, our results showed the LRP1B levels were significantly reduced in PC tissues and cell lines, indicating that down-regulation of LRP1B is at least partly due to increased miR-500 in PC. In our study, we headed to a new target, which is directly involved in many tumor developments. Exploring the specific and precise mechanism of miR-500 in prostate cancer is useful for precise medical targetted therapy. However, more animal experiments and clinically relevant detections are required further research to determine miR-500 and LRP1B.

In summary, we observed a strong down-regulation of miR-500 in PC and demonstrated a role for miR-500 as an important mediator of cell proliferation and invasion in PC. Our data indicate a suppressive role of miR-500 in PC development and may be a predictive biomarker and a novel therapeutic target for patients with PC, and may improve the prognosis of patients.

## Abbreviations

CDK2: cyclin-dependent protein kinase2
HC: hepatocellular carcinoma
LDL: low density lipoprotein
LRP1B: lipoprotein receptor-related protein 1B
NC: negative control
NF-κB: nuclear factor-kappaB
PC: prostate cancer
qRT-PCR: quantitative real-time PCR
